# Association between triglyceride glucose-waist height ratio index and incident thyroid nodules: a prospective cohort study

**DOI:** 10.1080/07853890.2026.2748230

**Published:** 2026-10-20

**Authors:** Ling Deng, Yi Liu, Yaojia Zhou, Xiaoxia Zheng, Min Ren, Min Zhang, Yang Liu

**Affiliations:** ^a^ Department of Health Management & Institute of Health Management, Sichuan Provincial People’s Hospital, University of Electronic Science and Technology of China, Chengdu, China; ^b^ Physical Examination Center, Yunyang County People’s Hospital, Chongqing, China; ^c^ Animal Experimental Center of West China Hospital, Sichuan University, Chengdu, China; ^d^ Department of Geriatric Endocrinology, Sichuan Provincial People’s Hospital, University of Electronic Science and Technology of China, Chengdu, China

**Keywords:** Triglyceride glucose-waist height ratio, thyroid nodules, insulin resistance, prospective cohort study

## Abstract

**Background:**

Insulin resistance is associated with thyroid nodule formation. The triglyceride-glucose (TyG) index is a widely recognized surrogate for insulin resistance, and its clinical value increases when integrated with adiposity indices. We tested whether an elevated triglyceride glucose-waist height ratio (TyG-WHtR) is independently associated with incident thyroid nodules.

**Methods:**

This prospective cohort study recruited 65,347 participants between January and December 2021, with follow-up surveys conducted between January and December 2025. We examined associations of baseline and cumulative TyG-WHtR quartiles with thyroid nodule occurrence using multivariable binary logistic regression. Restricted cubic spline (RCS) models tested nonlinear trends in the association between thyroid nodules and TyG-WHtR. Receiver operating characteristic (ROC) curves were generated to compare the area under the curve (AUC) across four metabolic metrics: baseline TyG, WHtR, TyG-WHtR, as well as cumulative TyG-WHtR, with thyroid nodule status as the outcome. We also conducted subgroup and sensitivity analyses, which collectively confirmed the robustness of our observed associations.

**Results:**

During a mean follow-up of 4.08 ± 0.27 years, 4,029 participants (15.8%) were diagnosed with thyroid nodules by 2025. In fully adjusted models, the highest versus lowest quartile of baseline TyG-WHtR showed an OR of 1.57 (95% CI: 1.22–2.02), and cumulative TyG-WHtR yielded an OR of 1.57 (95% CI: 1.25–1.96). Each 1-SD increment in baseline and cumulative TyG-WHtR corresponded to ORs of 1.15 (1.05–1.26) and 1.17 (1.07–1.27), respectively. RCS analyses confirmed linear dose-response relationships for both (P for nonlinearity > 0.05). ROC curve analysis demonstrated that cumulative TyG-WHtR had the highest AUC (0.539) for thyroid nodules.

**Conclusions:**

TyG-WHtR is independently associated with thyroid nodule risk, with cumulative TyG-WHtR showing a stronger association than baseline TyG-WHtR. Maintaining favorable TyG and waist circumference levels over time may reduce thyroid nodule risk in this Chinese population.

## KEY MESSAGES

Higher triglyceride glucose-waist height ratio is independently associated with an increased risk of thyroid nodules.

## Introduction

Thyroid nodules are a common endocrine disorder, most of which remain clinically silent throughout their course. With the widespread adoption of high-resolution ultrasonography, the incidental detection of thyroid nodules has become increasingly common over the past several years. The prevalence of thyroid nodules ranges from 19% to 68% in the general population [1,2]. A systematic review and meta-analysis published in 2022 reported an overall prevalence of thyroid nodules in the general population of 24.8% [3]. Most of these nodules are benign, but they carry a risk of malignancy, with approximately 10 − 15% being malignant [4–6].

The risk factors for thyroid nodules identified across numerous epidemiological investigations encompass demographic characteristics (age and sex), iodine nutrition, metabolic syndrome together with its constituent conditions (namely hyperinsulinemia, insulin resistance, hyperglycemia, dyslipidemia, hypertension, and obesity), in addition to serum thyroid-stimulating hormone (TSH) levels and radiation history [7–14]. A case-control investigation found that, relative to their nodule-free counterparts, patients with thyroid nodules had significantly higher Homeostasis Model Assessment of Insulin Resistance (HOMA-IR) values [15]. Recent evidence has highlighted the differential predictive capacity of various metabolic parameters for thyroid nodules, with central obesity and insulin resistance exerting the strongest influence, followed in descending order by dyslipidemia and hypertension [16]. Basic science research also supports that central obesity and insulin resistance independently promote the development of thyroid nodules and thyroid tumors [17–19].

Numerous methods assess insulin resistance, including direct and indirect approaches. Despite being regarded as the benchmark direct method for assessing insulin resistance, the hyperinsulinemic-euglycemic clamp (HEC) remains clinically underutilized owing to its high expense and procedural intricacy. Derived from fasting glucose and insulin concentrations, HOMA-IR is a commonly used indirect index that requires lower financial and operational resources than the clamp technique, facilitating its use in large-scale epidemiological investigations and clinical trials. Nevertheless, HOMA-IR use is limited in community clinical practice, as these facilities often cannot measure fasting insulin levels. Furthermore, HOMA-IR may not accurately reflect insulin resistance when pancreatic β-cell function is impaired. As a readily accessible surrogate, the triglyceride-glucose (TyG) index has shown significant concordance with HOMA-IR. Multiple studies have validated its accuracy in diagnosing insulin resistance and used it to identify it across diverse populations [20–22]. As a widely adopted anthropometric index for assessing central obesity, the waist-to-height ratio (WHtR) is computed by dividing waist circumference by the individual’s height. Compared with waist circumference, WHtR accounts for height and more accurately assesses visceral fat accumulation and metabolic risk. The triglyceride glucose-waist height ratio (TyG-WHtR), which integrates TyG with WHtR, further improves predictive performance and outperforms TyG alone in identifying high-risk subgroups. In cardiovascular research, the TyG-WHtR is closely associated with atherosclerotic cardiovascular disease [23,24]. A prospective longitudinal investigation has linked elevated TyG-WHtR values to the subsequent development of cardiometabolic multimorbidity, establishing its role as an independent risk indicator [25]. Recent studies have demonstrated that, even in euthyroid individuals, thyroid morphology and function parameters are correlated with body composition measures, and these associations may further translate into subclinical cardiovascular dysfunction [26–28].

Accumulating data have consistently indicated that metabolic dysregulation contributes to thyroid nodule risk, with particular emphasis on the roles of obesity, insulin resistance, and metabolic syndrome. However, most of these are cross-sectional studies that analyzed the association between thyroid nodules and indicators such as the TyG, waist circumference, and body mass index (BMI) at a single time point. Given that the association between the TyG-WHtR and thyroid nodules remains unexplored, we designed a prospective cohort study to address this gap. Because TyG-WHtR is a dynamic indicator, we hypothesized that cumulative TyG-WHtR is more strongly associated with incident thyroid nodules than single-measurement TyG-WHtR.

This study investigated the relationship between baseline TyG-WHtR and cumulative TyG-WHtR and the risk of incident thyroid nodules.

## Methods

### Study design and population

We recruited 65347 health screening participants who visited the Health Management Center of Sichuan Provincial People’s Hospital for the baseline survey between January and December 2021. We conducted follow-up surveys between January and December 2025.

We included 44180 participants who underwent thyroid ultrasound examination in both 2021 and 2025. Participants were excluded for the following reasons: (1) deficiency in fasting blood glucose (FBG), triglyceride (TG), height, or waist measurement in 2021 or 2025; (2) pregnancy or lactation in 2021 or 2025; (3) presence of liver or kidney dysfunction in 2021 or 2025; (4) history of malignancy; (5) history of thyroidectomy or radioactive iodine therapy; (6) history of thyroid disease other than thyroid nodules; (7) presence of thyroid nodules in 2021. Figure 1 illustrates the detailed inclusion and exclusion process.

**Figure 1.**
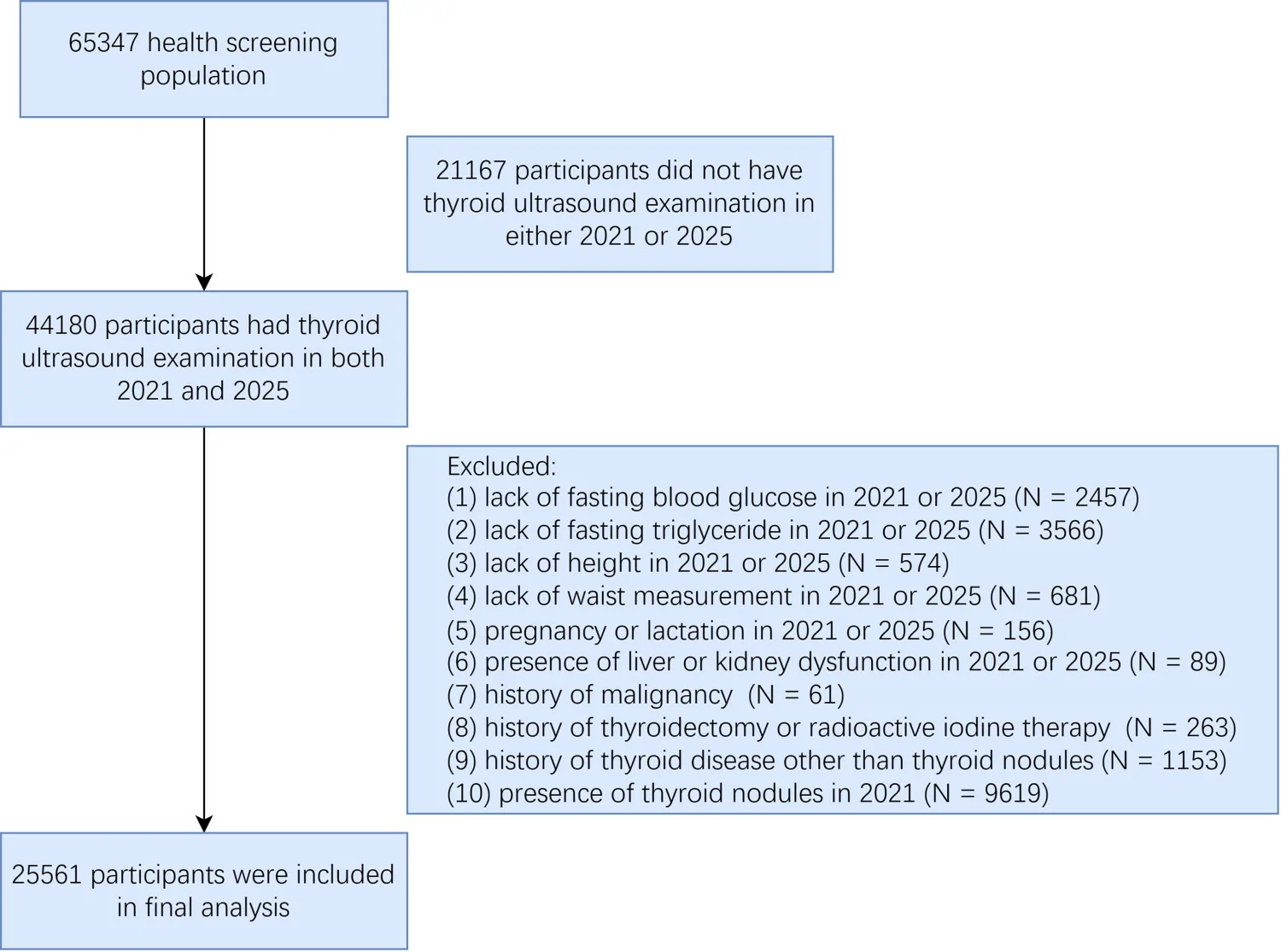
Flow chart of the study population.

This survey received ethical clearance from the Institutional Review Board of Sichuan Provincial People’s Hospital (approval No. 2020394, issued on July 29, 2020), and all procedures were performed in accordance with the ethical principles of the Declaration of Helsinki. All participants provided written informed consent before taking part in the study.

### Anthropometric measurements

Trained research staff performed physical examinations using calibrated instruments in accordance with standard operating procedures. We measured body height using an ultrasonic height gauge (OMRON HNN-318, Kyoto, Japan) with all subjects unshod and lightly clad. We considered a height variation greater than 10 cm between 2021 and 2025 an abnormal height measurement and excluded it from the analysis (*N* = 2). Waist circumference was recorded at the midpoint between the lower rib border and the upper iliac crest, with the tape kept parallel to the ground during measurement. To control for extreme values, waist circumference measurements that deviated from the mean by more than ±3 standard deviations (SD) were treated as outliers and omitted from the analytical sample (*N* = 13).

### Assessment of TyG-WHtR

We drew blood samples at two time points—the 2021 and 2025 visits—to determine fasting glucose and triglyceride levels. TyG-WHtR was calculated as ln [TG (mg/dL) × FPG (mg/dL)/2] multiplied by [waist (cm)/height (cm)], and cumulative TyG-WHtR was derived as the average of the 2021 and 2025 TyG-WHtR values multiplied by the time interval (2025 − 2021) [23,29].

### Assessment of thyroid nodules

We used an ultrasound system (Philips EPIQ 7, Philips Healthcare, Bothell, WA, USA) equipped with a linear-array transducer operating at 7.5–15 MHz. In both 2021 and 2025, we used the same ultrasound equipment, operating procedures, and assessment criteria. The ultrasound doctors were highly experienced and remained blinded to the metabolic data. A thyroid nodule is defined as a localized lesion with a maximum diameter of more than 2 mm that is located within the parenchyma and has a clear boundary. According to their composition, thyroid nodules can be classified as solid, cystic, mixed cystic and solid (containing both cystic and solid components), and spongiform nodules (composed of numerous small cystic spaces, with no solid tissue). When multiple thyroid nodules are present, consider the nodule with the highest TI-RADS category.

### Covariates

Covariates included demographic profiles, lifestyle habits, relevant disease history, physical measurements, and laboratory parameters (Table 1). Hypertension, diabetes, and dyslipidemia were defined based on the following criteria: (1) hypertension: systolic blood pressure (SBP) ≥140 mmHg, diastolic blood pressure (DBP) ≥90 mmHg, or self-reported history of hypertension; (2) diabetes: FPG ≥7.0 mmol/L, or self-reported history of diabetes; (3) dyslipidemia: total cholesterol (TC) ≥240 mg/dL, TG ≥150 mg/dL, low-density lipoprotein cholesterol (LDL-C) ≥160 mg/dL, high-density lipoprotein cholesterol (HDL-C) <40 mg/dL or self-reported history of dyslipidemia [29].

**Table 1.** Baseline characteristics of participants by baseline TyG-WHtR quartiles.

Characteristic	Total (*n* = 25561)	Baseline TyG-WHtR				P
		Q1 (*n* = 6390)	Q2 (*n* = 6391)	Q3 (*n* = 6390)	Q4 (*n* = 6390)	
Age, years	38 (32,48)	33 (29,38)	36 (31,45)	40 (33,50)	45 (36,53)	< 0.001
Sex, n (%)						< 0.001
Male	14689 (57.5)	1521 (23.8)	3090 (48.3)	4609 (72.1)	5469 (85.6)	
Female	10872 (42.5)	4869 (76.2)	3301 (51.7)	1781 (27.9)	921 (14.4)	
Smoking status, n (%)						< 0.001
Never	20202 (79.6)	5915 (92.9)	5448 (85.7)	4854 (76.6)	3985 (63.1)	
Others	5176 (20.4)	451 (7.1)	907 (14.3)	1483 (23.4)	2335 (36.9)	
Drinking status, n (%)						< 0.001
Others	16436 (64.6)	5403 (84.8)	4570 (71.7)	3632 (57.1)	2831 (44.5)	
Current	9021 (35.4)	966 (15.2)	1800 (28.3)	2727 (42.9)	3528 (55.5)	
Comorbidities, n (%)						
Hypertension	3382 (13.2)	130 (2.0)	430 (6.7)	932 (14.6)	1890 (29.6)	< 0.001
Diabetes	959 (3.8)	10 (0.2)	78 (1.2)	178 (2.8)	693 (10.8)	< 0.001
Dyslipidemia	8661 (33.9)	220 (3.4)	856 (13.4)	2620 (41.0)	4965 (77.7)	< 0.001
SBP, mmHg	117 (107,127)	109 (101,117)	113 (105,123)	119 (110,129)	126 (116,136)	< 0.001
DBP, mmHg	71 (65,79)	67 (61,72)	69 (63,76)	73 (67,80)	78 (71,86)	< 0.001
TC, mg/dL	182.1 (160.9,205.3)	168.6 (151.2,189.1)	178.7 (159.7,200.7)	187.2 (165.9,209.2)	195.9 (172.9,219.6)	< 0.001
LDL-C, mg/dL	101.7 (83.9,121.4)	87.4 (73.9,103.2)	100.9 (85.1,117.6)	109.4 (92.4,128.0)	111.4 (91.2,131.1)	< 0.001
HDL-C, mg/dL	54.5 (46.0,64.6)	65.0 (57.6,74.2)	57.6 (50.2,66.5)	51.0 (44.9,58.8)	45.6 (39.8,52.2)	< 0.001
HbA1c, %	5.36 (5.15,5.60)	5.20 (5.03,5.40)	5.30 (5.11,5.50)	5.40 (5.20,5.60)	5.55 (5.30,5.87)	< 0.001
TSH, mIU/L	1.90 (1.35,2.68)	1.86 (1.31,2.61)	1.91 (1.36,2.69)	1.91 (1.36,2.70)	1.94 (1.38,2.76)	< 0.001
FT3, pmol/L	4.38 (4.00,4.78)	4.18 (3.82,4.57)	4.32 (3.94,4.72)	4.45 (4.09,4.84)	4.56 (4.19,4.94)	< 0.001
FT4, pmol/L	12.78 (11.95,13.65)	12.93 (12.11,13.80)	12.84 (12.04,13.69)	12.72 (11.90,13.61)	12.59 (11.74,13.47)	< 0.001
TgAb, IU/mL	10.00 (10.00,10.00)	10.00 (10.00,12.04)	10.00 (10.00,11.26)	10.00 (10.00,10.00)	10.00 (10.00,10.00)	< 0.001
TPOAb, IU/mL	2.00 (2.00,3.00)	2.00 (2.00,3.37)	2.00 (2.00,3.30)	2.00 (2.00,2.82)	2.00 (2.00,2.66)	< 0.001
TyG-WHtR2021	3.94 (3.48,4.45)	3.23 (3.06,3.36)	3.71 (3.60,3.82)	4.18 (4.06,4.31)	4.82 (4.62,5.11)	< 0.001
TyG-WHtR2025	3.99 (3.54,4.48)	3.35 (3.14,3.59)	3.79 (3.56,4.05)	4.21 (3.94,4.48)	4.75 (4.42,5.11)	< 0.001
Cumulative TyG-WHtR	15.50 (13.68,17.54)	12.88 (11.98,13.68)	14.73 (13.90,15.50)	16.46 (15.56,17.35)	18.83 (17.73,20.26)	< 0.001
Thyroid nodules, n (%)	4029 (15.8)	873 (13.7)	1008 (15.8)	1007 (15.8)	1141 (17.9)	< 0.001

SBP, systolic blood pressure; DBP, diastolic blood pressure; TC, total cholesterol; LDL-C, low-density lipoprotein cholesterol; HDL-C, high-density lipoprotein cholesterol; HbA1C, glycated hemoglobin; TSH, thyroid-stimulating hormone; FT3, free triiodothyronine; FT4, free thyroxine; TgAb, anti-thyroglobulin antibody; TPOAb, anti-thyroid peroxidase antibody; TyG-WHtR, triglyceride glucose-waist height ratio.

### Data preprocessing

Table S1 presents the proportion of missing data for each variable. To minimize potential bias from missing data, we performed multiple imputation by chained equations (MICE). Before the formal analysis, we performed a multiple collinearity test for all candidate indicators (Table S2), with GVIF^(1/2DF) ≥ 2 indicating significant collinearity; here, GVIF denotes the generalized variance inflation factor, and DF refers to the degrees of freedom.

### Statistical analyses

We assessed the normality of continuous variables using the one-sample Kolmogorov‑Smirnov test. We presented continuous variables as mean ± SD or median (interquartile range [IQR]), and categorical variables as number (percentage). We categorized the participants into quartile-based groups (Q1–Q4) according to the baseline and cumulative TyG-WHtR, separately. We assessed the association between quartile categories and thyroid nodules using multivariable binary logistic regression. We constructed four multivariable models with progressive adjustment. Model 1 was adjusted for age and sex. Model 2 additionally included smoking and drinking status. Model 3 further accounted for hypertension, diabetes, and dyslipidemia. Model 4 was fully adjusted for all previous covariates plus SBP, DBP, TC, HDL-C, glycated hemoglobin (HbA1c), TSH, free triiodothyronine (FT3), free thyroxine (FT4), anti-thyroglobulin antibody (TgAb), and anti-thyroid peroxidase antibody (TPOAb). Because TC and LDL-C are strongly collinear, we did not include LDL-C as a covariate in Model 4. We also calculated P values for trends across categories and for each 1-SD increase in TyG-WHtR. We further used restricted cubic spline (RCS) regression to evaluate the dose-response relationship between baseline and cumulative TyG-WHtR and thyroid nodules. We used a systematic selection procedure for RCS modeling, sequentially evaluating models with 3, 4, and 5 knots. We selected the final model based on the Akaike information criterion (AIC), with a lower AIC value indicating a more parsimonious fit. To evaluate the diagnostic performance of baseline TyG, WHtR, TyG-WHtR, and cumulative TyG-WHtR for thyroid nodules, we plotted receiver operating characteristic (ROC) curves. We estimated the corresponding area under the curve (AUC) for each model. We conducted subgroup analyses to assess whether covariates (sex, smoking, drinking, hypertension, diabetes, and dyslipidemia) influenced the relationship between baseline/cumulative TyG-WHtR and thyroid nodules. As a sensitivity analysis, we re-ran all regression models using the complete-case dataset (*n* = 16,893), excluding observations with missing data.

For all statistical evaluations, we utilized R (version 4.5.3) and SPSS (version 26.0). A nominal two-sided P value of less than 0.05 was predetermined as the criterion for statistical significance.

## Results

### Baseline characteristics of participants

This study included 25561 participants without thyroid nodules at baseline. Table 1 presents the baseline characteristics of the study cohort. The median age (interquartile range, IQR) at baseline was 38 (32, 48) years, and 57.5% were males. The median TyG-WHtR (IQR) was 3.94 (3.48, 4.45) in 2021 and 3.99 (3.54, 4.48) in 2025, and the median cumulative TyG-WHtR (IQR) was 15.50 (13.68,17.54). Compared to the lowest quartile (Q1), participants in the upper three quartile groups (Q2–Q4) tended to be older and had a higher proportion of males. These groups exhibited elevated rates of smoking and drinking, along with greater burdens of hypertension, diabetes, and dyslipidemia. They had higher SBP, DBP, TC, LDL-C, HbA1c, TSH, FT3, and TyG-WHtR. They also had lower HDL-C and FT4 levels. Furthermore, Table 2 displays the baseline characteristics of the study population, stratified by quartiles of cumulative TyG-WHtR.

**Table 2.** Baseline characteristics of participants by cumulative TyG-WHtR quartiles.

Characteristic	Total (*n* = 25561)	Cumulative TyG-WHtR				P
		Q1 (*n* = 6390)	Q2 (*n* = 6391)	Q3 (*n* = 6390)	Q4 (*n* = 6390)	
Age, years	38 (32,48)	34 (29,39)	36 (31,46)	49 (33,50)	44 (35,52)	< 0.001
Sex, n (%)						< 0.001
Male	14689 (57.5)	1469 (23.0)	3099 (48.5)	4630 (72.5)	5491 (85.9)	
Female	10872 (42.5)	4921 (77.0)	3292 (51.5)	1760 (27.5)	899 (14.1)	
Smoking status, n (%)						< 0.001
Never	20202 (79.6)	5982 (93.9)	5454 (85.8)	4834 (76.2)	3932 (62.3)	
Others	5176 (20.4)	388 (6.1)	900 (14.2)	1511 (23.8)	2377 (37.7)	
Drinking status, n (%)						< 0.001
Others	16436 (64.6)	5458 (85.6)	4564 (71.7)	3634 (57.1)	2780 (43.8)	
Current	9021 (35.4)	915 (14.4)	1802 (28.3)	2731 (42.9)	3573 (56.2)	
Comorbidities, n (%)						
Hypertension	3382 (13.2)	191 (3.0)	469 (7.3)	959 (15.0)	1763 (27.6)	< 0.001
Diabetes	959 (3.8)	27 (0.4)	86 (1.3)	225 (3.5)	621 (9.7)	< 0.001
Dyslipidemia	8661 (33.9)	362 (5.7)	1034 (16.2)	2546 (39.8)	4719 (73.8)	< 0.001
SBP, mmHg	117 (107,127)	110 (102,119)	113 (105,123)	119 (110,130)	125 (115,136)	< 0.001
DBP, mmHg	71 (65,79)	67 (62,73)	69 (63,75)	73 (66,80)	77 (70,85)	< 0.001
LDL-C, mg/dL	101.7 (83.9,121.4)	88.9 (75.0,105.6)	100.2 (83.9,117.2)	109.0 (91.6,127.6)	110.2 (91.2,129.9)	< 0.001
HDL-C, mg/dL	54.5 (46.0,64.6)	65.4 (57.2,74.2)	57.6 (50.3,66.5)	51.4 (44.9,58.8)	45.6 (39.8,52.2)	< 0.001
TG, mg/dL	105.4 (72.6,160.3)	66.4 (53.1,84.1)	89.5 (70.9,117.8)	124.0 (94.8,164.7)	186.9 (136.4,268.4)	< 0.001
HbA1c, %	5.36 (5.15,5.60)	5.22 (5.06,5.40)	5.30 (5.11,5.50)	5.40 (5.20,5.60)	5.51 (5.29,5.80)	< 0.001
TSH, mIU/L	1.90 (1.35,2.68)	1.87 (1.33,2.62)	1.92 (1.36,2.72)	1.92 (1.35,2.71)	1.91 (1.37,2.68)	< 0.001
FT3, pmol/L	4.38 (4.00,4.78)	4.18 (3.83,4.55)	4.34 (3.96,4.75)	4.47 (4.10,4.85)	4.54 (4.16,4.92)	< 0.001
FT4, pmol/L	12.78 (11.95,13.65)	12.91 (12.10,13.78)	12.83 (12.01,13.71)	12.78 (11.94,13.63)	12.56 (11.72,13.42)	< 0.001
TgAb, IU/mL	10.00 (10.00,10.00)	10.00 (10.00,13.16)	10.00 (10.00,10.00)	10.00 (10.00,10.00)	10.00 (10.00,10.00)	< 0.001
TPOAb, IU/mL	2.00 (2.00,3.00)	2.00 (2.00,3.21)	2.00 (2.00,3.30)	2.00 (2.00,2.85)	2.00 (2.00,2.73)	< 0.001
TyG-WHtR2021	3.94 (3.48,4.45)	3.27 (3.07,3.48)	3.69 (3.50,3.90)	4.15 (3.93,4.38)	4.77 (4.49,5.09)	< 0.001
TyG-WHtR2025	3.99 (3.54,4.48)	3.31 (3.12,3.53)	3.75 (3.57,3.95)	4.19 (3.99,4.41)	4.79 (4.53,5.12)	< 0.001
Cumulative TyG-WHtR	15.50 (13.68,17.54)	12.57 (11.76,13.16)	14.62 (14.16,15.05)	16.45 (15.95,16.97)	18.98 (18.17,20.09)	< 0.001
Thyroid nodules, n (%)	4029 (15.8)	881 (13.8)	987 (15.4)	1048 (16.4)	1113 (17.4)	< 0.001

SBP, systolic blood pressure; DBP, diastolic blood pressure; TC, total cholesterol; LDL-C, low-density lipoprotein cholesterol; HDL-C, high-density lipoprotein cholesterol; HbA1C, glycated hemoglobin; TSH, thyroid-stimulating hormone; FT3, free triiodothyronine; FT4, free thyroxine; TgAb, anti-thyroglobulin antibody; TPOAb, anti-thyroid peroxidase antibody; TyG-WHtR, triglyceride glucose-waist height ratio.

### Odds ratios for incident thyroid nodules

During a mean follow-up of 4.08 ± 0.27 years, 4,029 participants (15.8%) were diagnosed with thyroid nodules by 2025. The number of participants with thyroid nodules across baseline TyG-WHtR quartiles (Q1-Q4) were 873, 1008, 1007, and 1141, respectively (Table 1). The results of logistic regression analyses for the associations of baseline TyG-WHtR and cumulative TyG-WHtR with thyroid nodules are shown in Table 3. Model 4, adjusted for all confounders, showed a significantly higher risk of thyroid nodules in the upper three baseline TyG-WHtR quartiles than in the lowest quartile. Specifically, the ORs (95% CI) for Q2, Q3, and Q4 were 1.37 (1.10–1.60), 1.35 (1.09–1.67), and 1.57 (1.22–2.02), respectively. Similarly, in the fully adjusted Model 4, when categorized by cumulative TyG-WHtR quartiles, the risk of thyroid nodules rose progressively from Q3 to Q4. Relative to Q1, the adjusted ORs (95% CI) were 1.47 (1.21–1.79) for Q3 and 1.57 (1.25–1.96) for Q4, with both showing significant differences. The P for trend across quartiles of both baseline and cumulative TyG-WHtR was less than 0.05. The fully adjusted analysis identified an independent positive association between baseline TyG-WHtR and incident thyroid nodule risk, with each SD increment yielding an OR of 1.15 (95% CI: 1.05–1.26). The association remained robust in the cumulative TyG-WHtR analysis, with each 1-SD increase corresponding to an OR of 1.17 (95% CI: 1.07–1.27). The RCS regression model revealed a linear increase in the risk of thyroid nodules with baseline TyG-WHtR in 2021 (P for nonlinearity = 0.108). Likewise, cumulative TyG-WHtR showed consistent findings (P for nonlinearity = 0.228) (Figure 2).

**Figure 2.**
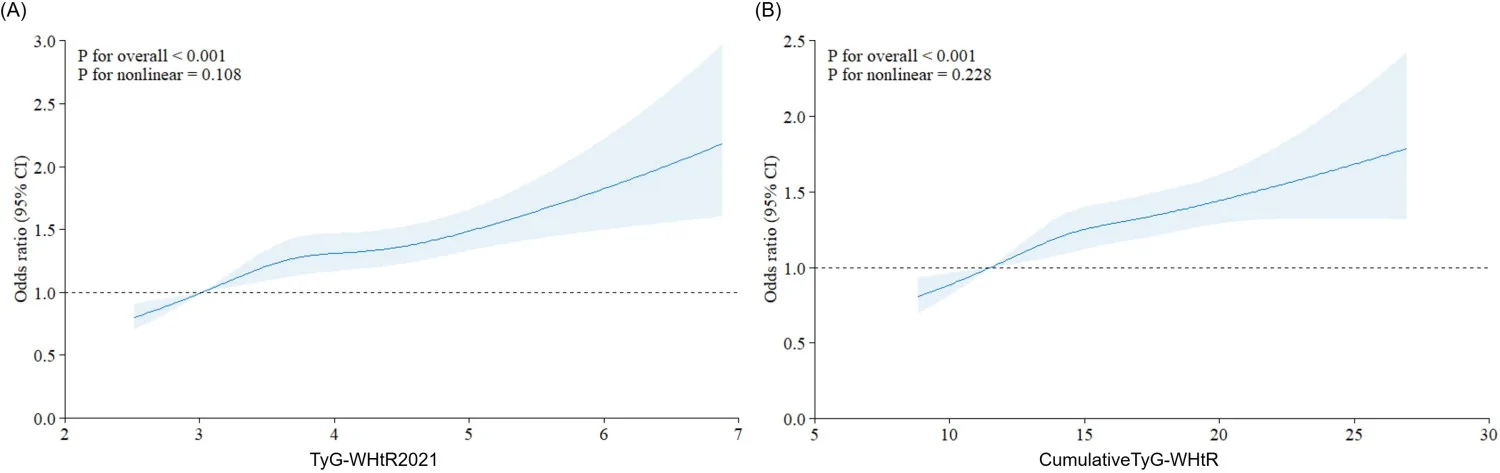
Restricted cubic spline models of the association of baseline TyG-WHtR (A) or cumulative TyG-WHtR (B) with thyroid nodules.

**Table 3.** Logistic regression models assessing thyroid nodule risk across categorical TyG-WHtR quartiles.

	Model 1	P	Model 2	P	Model 3	P	Model 4	P
**Baseline TyG-WHtR in 2021**								
Q2	1.20 (1.08–1.33)	<0.001	1.20 (1.09–1.33)	<0.001	1.21 (1.09–1.34)	<0.001	1.37 (1.10–1.60)	0.004
Q3	1.26 (1.13–1.41)	<0.001	1.26 (1.13–1.41)	<0.001	1.27 (1.14–1.43)	<0.001	1.35 (1.09–1.67)	0.007
Q4	1.49 (1.33–1.67)	<0.001	1.47 (1.30–1.65)	<0.001	1.48 (1.29–1.69)	<0.001	1.57 (1.22–2.02)	<0.001
Per SD	1.17 (1.12–1.22)	<0.001	1.16 (1.12–1.21)	<0.001	1.18 (1.12–1.24)	<0.001	1.15 (1.05–1.26)	0.003
**Cumulative TyG-WHtR (at the 2021 and 2025 time points)**								
Q2	1.20 (1.08–1.32)	0.001	1.20 (1.08–1.33)	0.001	1.20 (1.09–1.33)	<0.001	1.18 (0.98–1.41)	0.083
Q3	1.38 (1.24–1.54)	<0.001	1.39 (1.24–1.55)	<0.001	1.39 (1.25–1.56)	<0.001	1.47 (1.21–1.79)	<0.001
Q4	1.53 (1.36–1.71)	<0.001	1.51 (1.34–1.69)	<0.001	1.51 (1.33–1.72)	<0.001	1.57 (1.25–1.96)	<0.001
Per SD	1.18 (1.14–1.23)	<0.001	1.17 (1.13–1.22)	<0.001	1.18 (1.13–1.24)	<0.001	1.17 (1.07–1.27)	0.003

Data are presented as OR (95% CI). Compared with the Q1 group (reference). TyG-WHtR, triglyceride glucose-waist height ratio.

### Diagnostic efficacy of TyG-WHtR for thyroid nodules

Cumulative TyG-WHtR showed the strongest association with incident thyroid nodules, with the highest AUC (0.539, 95% CI 0.530–0.549) in ROC curve analysis, although the discriminatory ability was modest. By contrast, the AUCs for baseline TyG-WHtR, baseline WHtR, and baseline TyG were 0.534, 0.530, and 0.519, respectively (Figure 3).

**Figure 3.**
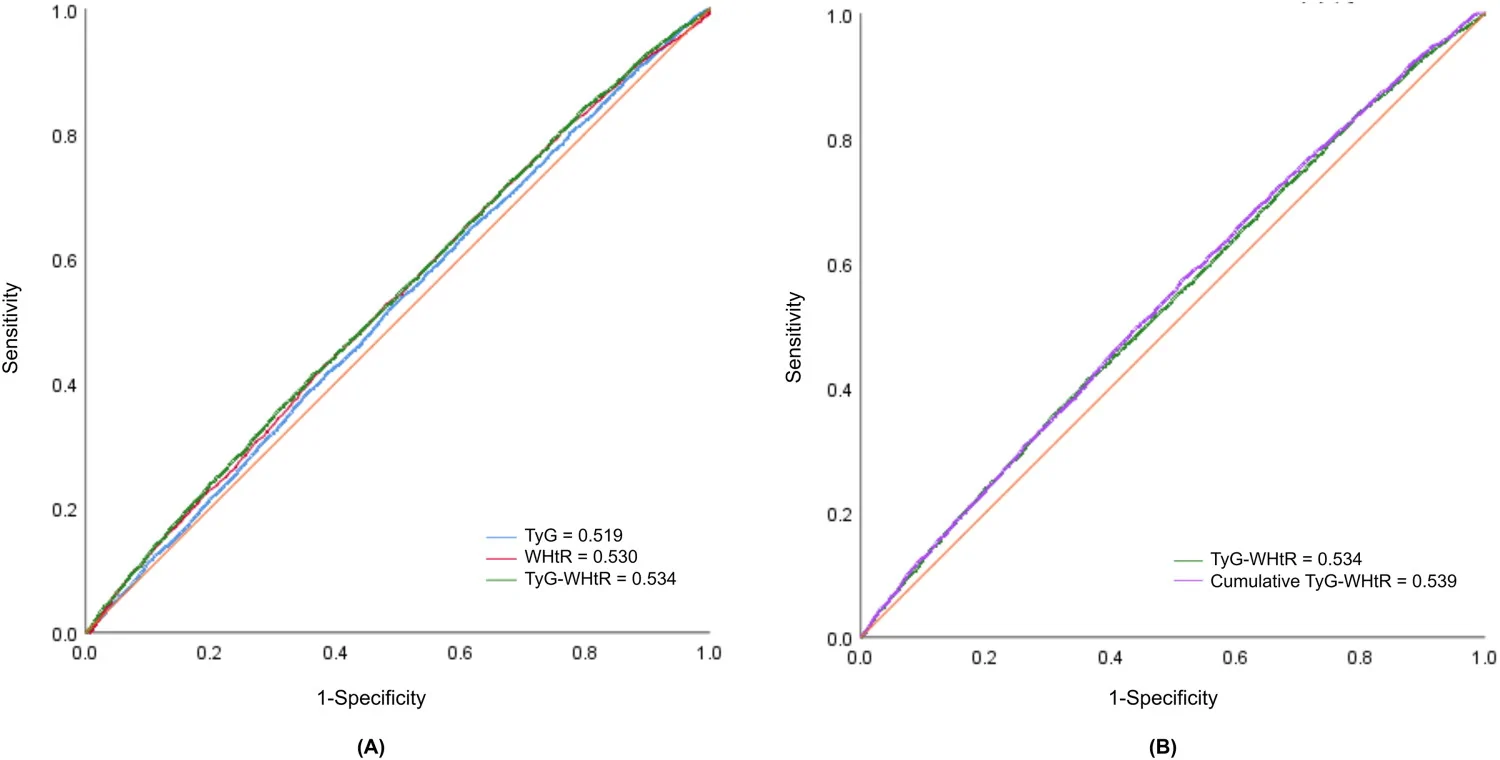
Receiver operating characteristic curve analysis of baseline TyG, WHtR, and TyG-WHtR for thyroid nodule prediction (A), and of cumulative TyG-WHtR for thyroid nodule prediction (B).

### Subgroup analyses and sensitivity analyses

To examine whether the associations varied across subgroups, we conducted stratified analyses for baseline TyG-WHtR and cumulative TyG-WHtR in relation to thyroid nodules. Tables 4 and 5 provide the detailed findings for these two indices, respectively. Significant interactions were observed for sex, smoking status, hypertension, diabetes, and dyslipidemia (all P for interaction < 0.05), but not for drinking status. Sensitivity analyses using the complete-case dataset yielded similar effect sizes and significance levels, confirming the robustness of our findings (Table S3).

**Table 4.** Association of baseline TyG-WHtR with thyroid nodule risk across diverse population strata.

Subgroup	Baseline TyG-WHtR			P for interaction
	Q2	Q3	Q4	
**Sex**				< 0.001
Male	1.21 (0.98–1.49)	1.53 (1.25–1.86)	2.00 (1.65–2.42)	
Female	1.46 (1.30–1.64)	1.68 (1.47–1.92)	2.07 (1.76–2.45)	
**Smoking status**				0.001
Never	1.21 (1.09–1.34)	1.21 (1.09–1.35)	1.34 (1.20–1.49)	
Others	1.38 (0.95–2.01)	1.56 (1.09–2.22)	2.17 (1.55–3.05)	
**Drinking status**				0.288
Others	1.26 (1.13–1.41)	1.27 (1.13–1.42)	1.47 (1.30–1.66)	
Current	1.14 (0.89–1.47)	1.34 (1.06–1.69)	1.67 (1.33–2.08)	
**Hypertension**				< 0.001
Present	1.89 (1.06–3.34)	1.74 (1.01–3.01)	1.82 (1.06–3.10)	
Absent	1.15 (1.04–1.27)	1.12 (1.01–1.24)	1.27 (1.15–1.42)	
**Diabetes**				< 0.001
Present	0.42 (0.11–1.65)	0.37 (0.10–1.37)	0.44 (0.12–1.59)	
Absent	1.18 (1.07–1.30)	1.18 (1.07–1.30)	1.32 (1.20–1.46)	
**Dyslipidemia**				
Present	1.01 (0.65–1.58)	1.25 (0.83–1.89)	1.43 (0.95–2.14)	0.03
Absent	1.22 (1.10–1.35)	1.20 (1.07–1.34)	1.58 (1.36–1.83)	

Data are presented as OR (95% CI). Compared with the Q1 group (reference). TyG-WHtR, triglyceride glucose-waist height ratio.

**Table 5.** Association of cumulative TyG-WHtR with thyroid nodule risk across diverse population strata.

Subgroup	Cumulative TyG-WHtR			P for interaction
	Q2	Q3	Q4	
**Sex**				< 0.001
Male	1.30 (1.05–1.61)	1.61 (1.32–1.97)	1.95 (1.60–2.37)	
Female	1.35 (1.20–1.51)	1.79 (1.57–2.05)	2.17 (1.84–2.56)	
**Smoking status**				0.003
Never	1.16 (1.05–1.29)	1.28 (1.15–1.42)	1.30 (1.16–1.45)	
Others	1.39 (0.94–2.07)	1.57 (1.08–2.28)	2.04 (1.43–2.93)	
**Drinking status**				0.442
Others	1.18 (1.06–1.32)	1.35 (1.21–1.51)	1.40 (1.24–1.58)	
Current	1.27 (0.98–1.64)	1.40 (1.10–1.78)	1.70 (1.35–2.18)	
**Hypertension**				< 0.001
Present	1.05 (0.68–1.63)	1.13 (0.75–1.69)	1.20 (0.82–1.77)	
Absent	1.13 (1.02–1.25)	1.19 (1.07–1.32)	1.22 (1.10–1.36)	
**Diabetes**				< 0.001
Present	1.25 (0.42–3.74)	1.49 (0.54–4.13)	1.20 (0.45–3.23)	
Absent	1.14 (1.02–1.25)	1.20 (1.09–1.32)	1.28 (1.16–1.42)	
**Dyslipidemia**				
Present	0.95 (0.67–1.34)	1.17 (0.85–1.61)	1.30 (0.95–1.76)	0.03
Absent	1.18 (1.07–1.31)	1.26 (1.13–1.41)	1.38 (1.19–1.59)	

Data are presented as OR (95% CI). Compared with the Q1 group (reference). TyG-WHtR, triglyceride-glucose-waist height ratio.

## Discussion

This prospective cohort study contributes novel evidence on the association between TyG-WHtR and thyroid nodules. The results of this study are summarized as follows: First, over the 4-year observation window, both baseline and cumulative TyG-WHtR levels conferred a significantly increased risk of incident thyroid nodules, independent of conventional confounders; Second, both forms of the TyG-WHtR showed a linear relationship with the risk of thyroid nodules; Third, the diagnostic ability of TyG-WHtR was superior to that of TyG or WHtR alone, and the cumulative TyG-WHtR had the best performance; Finally, subgroup analyses revealed significant interactions for sex, smoking status, hypertension, diabetes, and dyslipidemia.

Insulin resistance is a recognized risk factor for thyroid nodules, as demonstrated in numerous clinical studies [7,15, 16,30]. A cross-sectional, population-based study suggests that insulin resistance is an important determinant of the distribution, architecture, and vascular density of thyroid nodules, which might contribute to their growth and progression [31]. Insulin resistance is defined as decreased tissue responsiveness to insulin, which leads to compensatory hyperinsulinemia. Elevated insulin levels, in turn, increase bioavailable free insulin-like growth factor 1 (IGF-1). Downstream signaling cascades, including mTOR and MAPK, are activated when insulin and IGF-1 engage their specific receptors (insulin receptor and IGF-1 receptor, respectively) on thyroid follicular epithelial cells [7,16,32]. IGF-1 receptor activation has been proposed to play a critical role in the etiology of diverse neoplastic disorders, including thyroid nodules [33]. This activation drives thyroid cell proliferation, ultimately progressing to thyroid nodules or thyroid cancer. Animal studies also indicate that the insulin/IGF-1 family signaling can drive thyroid growth [34].

Multiple investigations have established a connection between obesity and thyroid nodules. Multivariate logistic regression analysis of cross-sectional data from 183,990 participants at a Chinese health examination center demonstrated that central obesity independently increased the risk of thyroid nodules [35]. Data from an observational study in Italy indicated that both obesity and diabetes contributed to thyroid nodule risk in females and aging populations. Additionally, the dominant nodule size was greater among obese individuals relative to their nonobese counterparts [36]. Ohira et al. conducted a prospective cohort study in Japan and identified overweight status as a risk factor for thyroid nodules in pediatric and adolescent populations [37]. A positive association between thyroid nodules and metabolic syndrome—as well as its components, including central obesity, hypertriglyceridemia, elevated blood pressure, and hyperglycemia—was confirmed by a meta-analysis of 15 studies with 468,845 subjects [11].

The pathophysiological processes linking obesity to thyroid nodules are not yet fully understood, as they involve multiple interacting mechanisms. Obesity is not merely increased body weight; it is a complex endocrine state that promotes thyroid pathology through the concerted action of multiple signaling pathways. First, chronic low-grade inflammation is a hallmark of obesity. In this inflammatory milieu, the secretion of the pro-inflammatory adipokine leptin is increased. Leptin promotes thyroid cell proliferation, growth, and angiogenesis by activating its receptors. Conversely, adiponectin secretion is reduced, despite its known anti-inflammatory, anti-proliferative, and anti-angiogenic properties [33]. Second, adipose tissue in obese individuals contains high levels of active aromatase, an enzyme that converts androgens to estrogens; these estrogens then promote vascular endothelial growth factor upregulation *via* estrogen receptor signaling pathways. This activation drives the progression of both benign and malignant thyroid nodules by promoting cell proliferation and angiogenesis [2,38,39]. Third, obesity leads to insulin resistance, which results in hyperinsulinemia. As a potent growth factor, insulin can directly stimulate thyroid cell proliferation and increase their sensitivity to TSH. In addition, insulin increases free IGF-1 levels. By activating IGF-1 receptors and the MAPK pathway, this signaling cascade promotes thyroid tissue hyperplasia and nodule formation [33,40]. Fourth, the chronic low-grade inflammation associated with obesity leads to various metabolic disorders, including hyperglycemia, hyperlipidemia, hyperinsulinemia, and insulin resistance. These conditions are closely linked to oxidative stress, which is characterized by an excess of harmful free radicals and reactive molecules. Excessive reactive oxygen species (ROS) not only cause cellular damage but also activate the MAPK and PI3K/AKT pathways, thereby driving normal thyroid cells toward nodular hyperplasia and tumorigenesis [38].

Based on the pathophysiological mechanisms discussed above, central obesity and insulin resistance interact to promote thyroid nodule development through multiple pathways. Central obesity and insulin resistance were the strongest predictors of thyroid nodules, with dyslipidemia and hypertension ranking next [16]. Therefore, we combined the TyG with the WHtR to derive TyG-WHtR, which reflects both insulin resistance and central obesity, and investigated its association with thyroid nodules. Compared with the TyG, the TyG-WHtR showed enhanced predictive performance for cardiovascular disease, as reported in several NHANES-based studies [25,41]. These results suggest that TyG-WHtR could be a more practical index for risk categorization and early detection of individuals susceptible to metabolic disorders. To the best of our knowledge, this is the first study to combine TyG and WHtR to evaluate their association with thyroid nodules.

Because the TyG-WHtR is time-varying, we categorized participants into quartiles based on baseline and cumulative TyG-WHtR values. Higher baseline TyG-WHtR quartiles were associated with a higher incidence of thyroid nodules. Compared with the lowest quartile, the highest TyG-WHtR quartile was associated with 1.57-fold increased odds of thyroid nodules. Analyses based on cumulative TyG-WHtR quartiles corroborated this pattern and showed a consistent trend. Individuals with greater cumulative TyG-WHtR exposure were at a higher risk of thyroid nodules. Our results align with Long Yan et al. whose national prospective cohort study showed that higher cumulative TyG-WHtR levels are independently associated with increased cardiometabolic multimorbidity [25]. Our findings underscore the clinical importance of monitoring thyroid nodule risk factors over time. To evaluate the consistency of TyG-WHtR across various clinical subgroups, we performed stratified analyses. The results showed that the association between TyG-WHtR and thyroid nodules was significantly modified by sex, smoking, hypertension, diabetes, and dyslipidemia, suggesting that the benefits of managing the TyG-WHtR vary across populations.

The current work has several strengths. First, it is the first prospective cohort study to examine the TyG-WHtR in relation to thyroid nodule risk. Additionally, its large cohort size enhances the statistical power. Second, the composite index TyG-WHtR reflects core components of metabolic dysfunction (insulin resistance and central obesity) and accounts for disease interactions and shared pathogenic mechanisms. Thirdly, we stratified participants into quartiles based on baseline TyG-WHtR and cumulative TyG-WHtR exposure. This allowed us to explore the association of both metrics with thyroid nodules. Furthermore, sensitivity analyses served as a validation tool to confirm the dependability of our primary estimates.

The present study also has several limitations. First, our sample consisted exclusively of Chinese participants recruited from a single health examination center, which may limit the extrapolation of our conclusions to broader or non-Chinese populations. Second, we diagnosed thyroid nodules based on ultrasound examination without pathological confirmation. Thus, the benign or malignant nature of the nodules could not be determined. Third, despite our efforts to account for major confounders, including age, sex, and lifestyle habits, we cannot exclude residual confounding from unmeasured factors such as medication use and dietary iodine. Future research should address these limitations by (1) conducting multi-center prospective cohort studies across diverse ethnic populations to validate our findings; (2) incorporating pathological confirmation *via* fine-needle aspiration biopsy to accurately classify nodule histology; and (3) collecting detailed data on dietary iodine intake and medication use to better control for residual confounding.

## Conclusions

Independent of other factors, TyG-WHtR was associated with the risk of thyroid nodules, and this association was more pronounced for cumulative TyG-WHtR than for baseline TyG-WHtR. Collectively, these findings indicate that maintaining favorable TyG and waist circumference over time is linked to a lower risk of thyroid nodules in this Chinese population.

## Supplementary Material

Supplementary materials.docx

## Data Availability

The datasets that support the findings of this study are available from the corresponding author upon reasonable request.
