# Supplementary material for: Association between triglyceride glucose-waist height ratio index and incident thyroid nodules: a prospective cohort study
Source: Ann Med. 2026 Oct 20;58(1):2748230. doi: 10.1080/07853890.2026.2748230 (PMC13686191; doi:10.1080/07853890.2026.2748230)
Supplement: Supplementary file 1 [file IANN_A_2748230_SM3390.docx]

**Table S1.** Distribution of missing data.

| Characteristics | No. of missing values | Percent (%) |
| --- | --- | --- |
| Age | 0 | 0 |
| Sex | 0 | 0 |
| Smoking status | 183 | 0.72 |
| Drinking status | 104 | 0.4 |
| Hypertension | 6 | 0.02 |
| Diabetes | 0 | 0 |
| Dyslipidemia | 0 | 0 |
| SBP | 26 | 0.1 |
| DBP | 26 | 0.1 |
| TC | 1 | 0.004 |
| LDL_C | 2 | 0.008 |
| HDL_C | 1 | 0.004 |
| HbA1c | 7852 | 30.7 |
| TSH | 8522 | 33.3 |
| FT3 | 8521 | 33.3 |
| FT4 | 8516 | 33.3 |
| TgAb | 8510 | 33.3 |
| TPOAb | 8510 | 33.3 |
| eGFR | 3 | 0.01 |
| ALT | 11 | 0.04 |
| AST | 3 | 0.01 |

SBP, systolic blood pressure; DBP, diastolic blood pressure; TC, total cholesterol; LDL-C, low-density lipoprotein cholesterol; HDL-C, high-density lipoprotein cholesterol; HbA1C, glycated hemoglobin; TSH, thyroid-stimulating hormone; FT3, free triiodothyronine; FT4, free thyroxine; TgAb, anti-thyroglobulin antibody; TPOAb, anti-thyroid peroxidase antibody; eGFR, estimated glomerular filtration rate; ALT, Alanine Aminotransferase; AST, Aspartate Aminotransferase; No, number

**Table S2**. Collinearity statistics.

| Variable | GVIF | DF | GVIF^(1/(2*Df)) |
| --- | --- | --- | --- |
| Age | 1.408 | 1 | 1.187 |
| Sex | 1.939 | 1 | 1.392 |
| Smoking status | 1.357 | 1 | 1.165 |
| Drinking status | 1.550 | 1 | 1.245 |
| Hypertension | 1.759 | 1 | 1.326 |
| Diabetes | 1.778 | 1 | 1.333 |
| Dyslipidemia | 1.896 | 1 | 1.377 |
| SBP | 3.070 | 1 | 1.752 |
| DBP | 2.699 | 1 | 1.643 |
| TC | 6.483 | 1 | 2.546 |
| LDL-C | 5.122 | 1 | 2.263 |
| HDL-C | 2.572 | 1 | 1.604 |
| HbA1c | 1.998 | 1 | 1.414 |
| TSH | 1.086 | 1 | 1.042 |
| FT3 | 1.303 | 1 | 1.142 |
| FT4 | 1.273 | 1 | 1.128 |
| TgAb | 1.278 | 1 | 1.130 |
| TPOAb | 1.308 | 1 | 1.144 |
| TyG-WHtR2021 | 2.864 | 1 | 1.692 |

SBP, systolic blood pressure; DBP, diastolic blood pressure; TC, total cholesterol; LDL-C, low-density lipoprotein cholesterol; HDL-C, high-density lipoprotein cholesterol; HbA1C, glycated hemoglobin; TSH, thyroid-stimulating hormone; FT3, free triiodothyronine; FT4, free thyroxine; TgAb, anti-thyroglobulin antibody; TPOAb, anti-thyroid peroxidase antibody; TyG-WHtR, triglyceride glucose-waist height ratio.

Table S3. Logistic regression analysis for the association between different quartiles of TyG-WHtR and thyroid nodules in subpopulations of 16893 participants with complete data.

|  | **Model 1** | | **Model 2** | | **Model 3** | | **Model 4** | |
| --- | --- | --- | --- | --- | --- | --- | --- | --- |
|  | OR(95%CI) | P value | OR(95%CI) | P value | OR(95%CI) | P value | OR(95%CI) | P value |
| Baseline TyG-WHtR in 2021 | |  |  |  |  |  |  |  |
| Q1 | Reference |  | Reference |  | Reference |  | Reference |  |
| Q2 | 1.27(1.12-1.43) | <0.001 | 1.27(1.12-1.43) | <0.001 | 1.27(1.12-1.44) | <0.001 | 1.33(1.10-1.60) | 0.004 |
| Q3 | 1.28(1.12-1.47) | <0.001 | 1.28(1.12-1.47) | <0.001 | 1.31(1.14-1.51) | <0.001 | 1.35(1.09-1.67) | 0.007 |
| Q4 | 1.57(1.36-1.81) | <0.001 | 1.55(1.34-1.79) | <0.001 | 1.61 (1.37-1.90) | <0.001 | 1.57(1.22-2.02) | <0.001 |
| P for trend |  | <0.001 |  | <0.001 |  | <0.001 |  | 0.001 |
| Per SD | 1.18(1.12-1.24) | <0.001 | 1.17(1.11-1.23) | <0.001 | 1.21(1.14-1.28) | <0.001 | 1.15(1.05-1.26) | 0.003 |
| Cumulative TyG-WHtR | |  |  |  |  |  |  |  |
| Q1 | Reference |  | Reference |  | Reference |  | Reference |  |
| Q2 | 1.23(1.08-1.39) | 0.001 | 1.22(1.08-1.39) | 0.001 | 1.23(1.09-1.39) | 0.001 | 1.18(0.98-1.41) | 0.083 |
| Q3 | 1.45(1.28-1.66) | <0.001 | 1.45(1.27-1.65) | <0.001 | 1.47(1.29-1.69) | <0.001 | 1.47(1.21-1.79) | <0.001 |
| Q4 | 1.61(1.40-1.85) | <0.001 | 1.59(1.38-1.83) | <0.001 | 1.64(1.40-1.91) | <0.001 | 1.57(1.25-1.96) | <0.001 |
| P for trend |  | <0.001 |  | <0.001 |  | <0.001 |  | 0.001 |
| Per SD | 1.19(1.14-1.25) | <0.001 | 1.18(1.13-1.24) | <0.001 | 1.20(1.14-1.27) | <0.001 | 1.17(1.07-1.27) | 0.003 |

TyG-WHtR, triglyceride glucose-waist height ratio; OR, odds ratio; CI, confidence interval; SD, standard deviation
